# Taking Pause: A COVID-19 Student Reflection on Global Health Research Opportunities, Training, and Institutional Reform

**DOI:** 10.3389/fsoc.2022.768821

**Published:** 2022-01-20

**Authors:** Casey Chu, Gianna Griffin, Joseph L. Williams

**Affiliations:** ^1^ Yale School of Public Health, Yale University, New Haven, CT, United States; ^2^ Yale College, Yale University, New Haven, CT, United States; ^3^ Yale Institute for Global Health, Yale University, New Haven, CT, United States

**Keywords:** COVID-19, student research, global health, training, student reflection, ethics education and training

## Abstract

Restrictions to research due to COVID-19 have required global health researchers to factor public health measures into their work and discuss the most ethical means to pursue research under safety concerns and resource constraints. In parallel, global health research opportunities for students have also adapted to safety concerns and resource constraints. Some projects have been canceled or made remote, but inventively, domestic research opportunities have been created as alternatives for students to continue gaining global health learning competencies. Knowing the ethical challenges inherent in short-term student global health research and research in strained health systems, it is intriguing why these safer alternatives were not previously pervasive in global health education. This paper provides perspectives from students training at academic institutions in the US on how COVID-19 disrupted student research and what can be learned from the associated shifts in global health research. Additionally, the authors take this opportunity to advocate for academic institutions from high-income countries to reflect on long-standing global health research conventions that have been perpetuated and bolster training for students conducting global health research. The authors draw on their experiences, existing literature, and qualitative interviews with students who pursued global health research during COVID-19.

## Introduction

COVID-19 has threatened public health practice and research. Under pressure from this global public health emergency, several global disparities and structural injustices have been illuminated, notably access to adequate healthcare and essential treatments during this time (Wouters et al., 2021). Disruptions caused by COVID-19 have provided institutions and researchers alike an opportunity to reconsider the ethics of their public health services and research. Specifically, travel and gathering guidelines aimed at preventing viral transmission and reducing strain on health systems with dwindling resources have pushed research institutions and research ethics boards to implement policies that restrict activities, putting research on hold (Lumeng et al., 2020; Nature Medicine, 2020; Leal Filho et al., 2021). As a result, global health researchers have been required to factor public health measures into their work and discuss the most ethical means to pursue research under safety and resource constraints (Yeoh and Shah, 2020). While these concerns and constraints were already prevalent before the pandemic, it is curious why this level of scrutiny had not been applied before the pandemic, especially when many projects are conducted in low-resource environments and involve unequal partnerships that limit communities’ ownership and input (Bukusi et al., 2018; Pratt, 2020, 202).

Student-led global health research has adapted as well. Many student projects have been canceled, and research programs have created local research opportunities as alternatives for students to experientially gain global health learning competencies, such as the ability to adapt to low-resource settings, be socio-culturally aware, and apply social justice principles to practice (Jacobsen et al., 2019). Considering the well-known ethical challenges inherent in short-term experiences in global health and global health research, one may question why these safer alternatives, motivated by COVID-19, were not already the status quo (Tryon et al., 2008; Standish et al., 2014). The emergence of these alternatives is evidence that typical global health competencies do not preclude local opportunities as ways to achieve them.

This paper provides our perspectives, as undergraduate and graduate students from an academic institution in the US, on the impact of COVID-19 on student global health research and lessons learned. We argue that the alternatives to student-led global health research and heightened awareness of ethical conduct in global health research during COVID-19 should be maintained post-pandemic. Moreover, we suggest that academic institutions improve global health research training and commit to guidelines for ethical global health education. We primarily draw on our own experiences and literature on global health *research* student ethics, which is lacking; thus, we included studies related to *professional* student global health experiences. We also use excerpts from qualitative interviews conducted with students involved in human-subjects global health research projects during COVID-19 from a separate but related study led by one of the authors. Lastly, we end with a list of recommendations for academic institutions.

Interviews for this reflection received institutional review board (IRB) exemption by the Yale University IRB. Eligible students were enrolled in a US institution, over 18 years of age, and conducted global health human subjects research on domestic or international populations. Participants were required to have IRB approval and operate via a remote platform during the COVID-19 pandemic (post-March 2020) to be considered for inclusion. Thirty-nine students showed interest in participating in a 30 min qualitative interview, and eleven eligible participants were interviewed throughout 2021. Most informants were graduate students (e.g., MPH, MA, MS), but some undergraduate and professional students (e.g., medical, physician associate) participated. Gender was equally distributed. Most participants identified as *White*, and several identified as *African American/Black, Asian, Hispanic/Latino*, or *Other.* Student projects were conducted remotely in Asia, the Pacific Islands, Eastern Africa, the Middle East, South Korea, Russia, and the US. Examples of research topics included refugees’ health during COVID-19, physician perspectives on interventions like Pre-Exposure Prophylaxis (PrEP), and mental health.

### Student Global Health Research During COVID-19

In parallel with research in most fields, the nature of global health research opportunities available to students has also changed dramatically due to COVID-19. Many existing opportunities were canceled or adapted into remote global health research. Our interviews revealed that remote projects during this time were challenging pursuits (Table 1). When asked about the general challenges of remote research, students described how typical research tasks took more time, involved technological barriers, and introduced issues with relationship building. When asked about the ethical challenges of remote research, students described issues regarding informed consent, privacy, ensuring ethical conduct of study team members, and dealing with sensitive conversations with study participants.

**TABLE 1:** Examples of challenges students faced in global health research during COVID-19.

	Challenge	Example quote
General and logistical challenges	Time expenditure	*“[I would emphasize] repetition with your team if you’re going to do a purely remote project where you’re never going to meet the people on your team…you need to put extra time into the things that you already would have done on the ground”*
	Technological barriers	*“The Wi-Fi wasn’t great. So I would say for 75% of the interviews we couldn’t do the video on the whole time… and then there were, you know, some participants usually older participants…and they really struggled with zoom…”*
	Building relationships	*“I felt like my community partners were like, way more hands off and they would have been if I was in person. And I think that’s just because it’s harder to do…relationship building and building that trust when you’re far away. And I think certainly… we’ve done the best we can, given that it’s remote.”*
Ethical challenges	Informed consent	*“…if you were in person with someone. I think you might be able to gauge a little bit easier, how much they’ve read the consent form and how much you know they understood of the consent form…the remote platform it made it harder to gauge that”*
	Privacy	*“I just can’t always have a private space, and there were, you know, a lot of instances where I had to do the interview and my partner is like in the same room… I even took some calls in my bathroom… I did it because I thought like this is ethically the best situation but it’s not ideal…potential[ly] having someone overhear the conversation”*
	Ensuring ethical conduct of entire study team	*“… the biggest one is that you again you’re not as not being the person on the ground, you’re having to put a great deal of trust inside of the remainder of your team to and to make sure that they’re following [ethical] guidelines”*
	Sensitive conversations with study participants	*“… those interviews, they got pretty dark, talking about you know family members who’ve died from suicide friends who died from suicide their own depressive thoughts which was certainly weren’t questions that I probe for, but they just came up kind of organically and unprompted…I reached out to one of my mentors saying like, okay, technically the IRB has like approved us to keep going, but I feel a little bit uncomfortable, like interviewing these young adults and having a really challenging difficult conversation. And then just clicking a button to end the zoom call and then I have no idea where they are, how they’re doing.”*

Global health research is typically health research on populations from another country (usually low-and-middle-income countries), but our interviews with students showed that COVID-19 has perhaps broadened it to Koplan et al.’s definition of encompassing research that “improves health and achieves health equity for all people worldwide” (Koplan et al., 2009). Interestingly, we found that *domestic* research on a local population was acknowledged as a student global health research opportunity. One of the authors was awarded a student fellowship to conduct domestic research on refugee and asylum-seeking populations in their local community during COVID-19. Domestic research on local populations had never been permitted in the history of the fellowship, which kept a conservative definition of global health research that required students to travel internationally and conduct work on global populations. The author recounts their experience in the following reflection:

“*The COVID-19 pandemic was the catalyst for my grant-awardees’ opening criteria to domestic global health populations. I saw a need in my community, but due to geographic location, a previous application would have been rejected. I am actually very happy to be working domestically. I not only gain knowledge of global health and the skill-set that comes from navigating barriers unique to global health, but I have the opportunity to continue working with this global health population well after my research is done and/or to adjust my research as needed. I do not have as stringent time constraints as I would if I traveled abroad.*”

This example demonstrates how existing conventions have been transformed for the benefit of students but also for communities that benefit from such research. Importantly, it highlights the value of local research, echoing the pervasive view that global health includes local health and that global health education should include local and global contexts (Rowthorn, 2016; Wees and Holmer, 2020).

Local opportunities occur in a familiar healthcare system and reduce cultural and language differentials (DeCamp et al., 2018). They also allow students to remain engaged for extended periods, ensuring proper knowledge translation and communication of findings back to the community. This would combat common critiques that global health research experiences for students produce unsustainable outputs and inadequate benefits, among many others (Provenzano et al., 2010; Bauer, 2017).

Global health research can offer irreplaceable experiences that will equip students with comprehensive competencies and contextualize the concepts (e.g., social determinants of health, capacity building) they learn about in the classroom (Jogerst et al., 2015; Kalbarczyk et al., 2020). Students develop adaptability, problem-solving skills, and cultural competency, humility, and sensitivity, preparing them to work effectively in diverse communities. Certainly, these competencies can be gained in domestic research (Jogerst et al., 2015; Jacobsen et al., 2019; Rabin et al., 2021). Moreover, eliminating domestic research opportunities for global health students would be a disservice to local communities that could benefit significantly from research conducted by students who are motivated by a sense of goodwill and an interest in generating evidence that will reduce the health inequities. COVID-19 has shown us that when international host communities face health crises that make research ethically challenging, we should legitimize domestic research opportunities as ways for students in global health to fulfill program requirements and competencies.

### Ethics in Global Health Research Training

Whether research is on international or domestic populations, academic institutions must ensure that global health research ethics (GHRE) is properly taught to students pursuing global health research. GHRE encompasses the governance of research practices, ethical principles, and moral integrity in global health research. It is a necessary safeguard for global health research because harm still occurs even when well-intentioned researchers follow the proper ethical review processes and have well-established research partnerships (Yassi et al., 2013; De Visser et al., 2020; Nyirenda et al., 2020).

Typically, student-led global health research projects have focused on students’ needs and learning priorities. However, with GHRE principles integrated into research training, we could bring much-needed attention to host communities’ needs and priorities in research post-COVID-19. These principles include the basic bioethical principles (autonomy, non-maleficence, beneficence, and justice) and those frequently identified in the global health literature (e.g., Lasker et al.’s six core principles such as “host partner defines the program and needs of host community”) (Lasker et al., 2018). Understanding GHRE is necessary for students who pursue short-term experiences and have little exposure to different cultures and experience working in low-resource settings (Pinto and Upshur, 2009). Preparing students for ethically challenging situations will equip students with the tools and resources necessary to navigate ethical dilemmas without doing further harm.

Even though discussions about GHRE are making their way into academic institutions, applying GHRE in practice has been less effective in preparing students for ethical dilemmas (Standish et al., 2014; Rowthorn et al., 2019; Chiumento et al., 2020; Wright, 2020). Currently, most training workshops for students pursuing global health research projects are brief, covering only logistics and safety, and seldom incorporate training on dealing with ethical issues despite students being eager to learn how to be an ethical researcher:

“*I was grateful for the fact that like we had training because… at my previous institution… we didn’t have like pre like placement training, and I remember…I was like very vocal for advocating that we needed something like that because I think there is, you know, potential danger for people, you know, to just be put in those kind of situations.*”

“*I think that would have been helpful I feel like me having training in the local culture would have been beneficial no matter what… there’s a lot of… implicit social norms that kind of need to be unpacked…because then the folks that I’m speaking to will actually like unpack these concepts that might not [need to] be unpacked [if it were with someone from the community]*”

Critics have questioned students’ motivations, calling out their ignorance; however, with proper guidance and training, students stand to gain an ability to critically appraise their research with an ethical lens. When incorporated, GHRE training can prompt students to reflect on their role as a researcher:

“*…it really challenged us to be reflective about, what it means to be an ally, what does it mean to partner with communities in a truly ethical and non-tokenizing manner, [in turn] causing us to confront who benefits from the research. If the community isn’t benefiting, then it’s probably not worth doing.*”

“*[I asked myself], am I benefiting because I get papers, because I get promotions and because I get to travel to a cool place? Or, is the community truly benefiting? [The course allowed more] thinking about [questions such as] if the community isn’t benefiting, then it’s probably not worth doing.*”

“*I think like having that training, even though it was really intensive just completely transformed how I see myself and prompted me to be a lot more reflective about my positionality and how, even if I don’t mean to, some of my actions could be causing more harm than good.*”

Training should teach students to be aware of cultural differences, local laws and standards regarding planned activities, the history of the country, local health needs, and general global health inequities that may exist in research settings (Lasker et al., 2018; Kalbarczyk et al., 2020). It should also foster research integrity from study ideation to dissemination of findings (Bukusi et al., 2018). Lastly, students should receive both conceptual and practical (e.g., case-based) training that motivates ethical reflection and gives students tools to deal with issues (Mulhearn et al., 2017; DeCamp et al., 2018). This is vital because, despite receiving some form of training and having the ability to *identify* ethical issues, students still report being hesitant to seek help, feeling ill-equipped to address ethical issues, and having trouble communicating with study participants and local project teams (Standish et al., 2021).

### Institutional Reforms

From our perspective, as students studying global health at an academic institution in the US, we believe that direct improvements to training students alone will not promote sustainable shifts towards ethical student-led global health research. Structural changes to the academic context, especially in high-income countries like the US, through policy and practice are necessary. There are numerous competent global health education guidelines that academic institutions from high-income countries can draw from. The Working Group on Ethics Guidelines for Global Health Training (WEIGHT) ’s best-practice guidelines for training experiences in global health can be used to clarify and improve relationships between stakeholders (Crump and Sugarman, 2010). These include specific guidelines for sending institutions, host institutions, trainees, and mentors and emphasize strengthening student and mentor relationships with resources that can facilitate the monitoring and resolution of potential ethical concerns in research settings. Objectives for training students can be informed by Pinto and Upshur (2009)’s four ethical principles of humility, introspection, solidarity, and social justice for students and trainees to apply to global health work. Lastly, the Brocher Declaration, which many academic institutions and research centers have signed, contains guiding principles for global health work that can aptly apply to global health education and research, especially concerning building partnerships and the welfare of local communities involved (Brocher Foundation, 2020). This declaration can be broadly applied to ensure ethical global partnerships, the foundation of many short-term student global health research projects.

Finally, in discussing institutional reform, we would be remiss if we ignored academic institutions’ role in influencing students’ intentions to pursue global health research. Many academic institutions, particularly admissions committees, commonly deem international experiences as “enriching” experiences that support a global health or health-related career (Shah et al., 2019). As students are still learning, growing, and finding footing in global health, these expectations alter their intentions and value judgments when encountering ethical issues in their projects, despite most having a sense of goodwill. Consequently, instead of their goal being to support and uplift a community, it shifts towards impressing an admission committee. When institutions promote international global health research opportunities among their students, it also furthers this frame of mind. We want to bring this rarely discussed issue to light through our unique perspective. To rectify this issue, institutions should discourage such biases in academic programs and communicate these values appropriately to applicants.

### COVID-19 as an Opportunity for Improvement

Despite global health research ethics existing as a prominent field of study at many leading academic institutions, it is understandable why global health research ethics may be inadequately applied to practice. The movement towards discovering ethical harms in global health may outpace institutions’ capacity to reform antiquated structures and processes. Decentralized leadership among academic institutions and research centers also makes instigating a concerted effort to dismantle existing barriers to ethical research practices cross-sectionally challenging.

COVID-19 and its associated disruptions have provided an opportunity for inventive forms of virtual research and study. Travel restrictions and heightened COVID-19 risks worldwide have significantly reduced in-person student research experiences in global settings. However, many student research methods have emerged in response to the pandemic, such as virtual interviews, telehealth, and social media research (Hou et al., 2020). These innovative research methods can make research capacity more accessible in global settings during COVID-19 and in the future.

Additionally, the associated attention to ethically permissible research presents an opportunity to reflect and plan for reforms. As such, we offer a set of recommendations that are informed by the student interviews, literature, and guidelines presented in this reflection (Table 2).

**TABLE 2:** Recommendations.

1. Offer and encourage *local* global health research opportunities
2. Invest in GHRE training for students^a^
3. Develop training standards for global health research students and ensure all global health mentors are prepared to help students apply them to practice^b^
4. Advocate for elevated review processes for all student research projects (in addition to IRB)^c^
5. Develop mechanisms to regularly review current and new global health research partnerships to ensure equal participation, resolve and discuss any past/existing/potential ethical issues, and plan ways to strengthen partnerships^d^
6. Create services for students and mentors to appraise and resolve potential ethical issues^e^
7. Address biases in school admissions processes that disproportionately favor global health experiences abroad

a This can involve dedicating funds to run workshops, hire global health ethics faculty, or sponsor students to attend external training. Training should be created in collaboration with host institutions (Crump and Sugarman, 2010; Cherniak et al., 2017).

b Mentors of students pursuing global health research should be trained on these standards to model ethical research practices and help students resolve potential ethical issues in their work.

c For example, additional checklists to assess students’ readiness to conduct research (e.g., to ensure they have appropriate skills and knowledge for their project) and accountability check-ins to ensure the ethical conduct of research (Shah et al., 2019; Rattani and Hyder, 2021).

d These underlying partnerships that students’ research experiences are built on should be scrutinized and strengthened to minimize ethical dilemmas.

e For example, ethics consultation services or specific procedures for researchers to follow to resolve potential ethical issues.

## Discussion

While the COVID-19 pandemic has complicated global health research, it has also provided space to reflect on issues once taken at face value.

The disruption of COVID-19 on student research is unavoidable and expected since these are short-term and often low-priority endeavors. However, for students with time-limited degrees and breaks from classes, these would have been significant opportunities for growth and essential for a career in global health. By creating domestic opportunities, academic institutions are rightly asking how to continue fostering global health educational competencies to accommodate new barriers (i.e., travel restrictions, safety concerns, and resource constraints). **However, since some of these barriers existed before COVID-19, why hadn’t academic institutions encouraged students to conduct domestic research opportunities before?** This is the question we started with, which led us to reflect on the systems and conventions of global health that institutions are perpetuating with such practices. It also made us contemplate the type of global health researchers that academic institutions are fostering with such practices.

The necessary reforms to global health research and education outlined in this paper are not issues students can rectify on their own. We believe there are ample opportunities for academic institutions to take charge. The main reforms mentioned, such as creating more domestic research opportunities for students and integrating more GHRE into training, would signal an institution’s commitment to global health that includes local communities. These actionable recommendations acknowledge that all health inequities deserve attention, no matter which part of the world they are from. Such improvements would not only have a ripple effect in fostering the development of ethical global health practitioners but would inevitably contribute to decolonizing global health research and education.

We end this reflection with a fitting quote by Arundhati Roy, which we direct at academic institutions that are pausing, reflecting, and improving during this time:

“*Historically, pandemics have forced humans to break with the past and imagine their world anew. This one (COVID-19) is no different. It is a portal, a gateway between one world and the next. We can choose to walk through it, dragging the carcasses of our prejudice…and dead ideas…Or we can walk through lightly, with little luggage, ready to imagine another world. And ready to fight for it*” (Roy, 2020).

## Data Availability

The original contributions presented in the study are included in the article/Supplementary Material, further inquiries can be directed to the corresponding author.
